# Journal of Proceedings of the Michigan Medical Association for the Years 1849 and 1850, Vol. 1

**Published:** 1850-07

**Authors:** 


*Journal of Proceedings of the Michigan Medical Association for the years* 1849 *and* 1850, *vol.* 1. Jackson, Michigan, 1850.

The Michigan Medical Association was first organized under a legislative act, in 1819, while Michigan was under a territorial government. Its legal existence was continued after the organization of a *State* government, until, in 1844, all laws relating to the medical profession were repealed. In 1846, when the statutes were revised, the Society was revived with its former powers and immunities. A satisfactory reorganization, however, was not effected until January, 1849, at which time a convention met at Jackson, the result of which was the establishment of the association on a firm basis. The committee on publication state, that "the only object of those engaged in this undertaking has been to contribute their share to the effort now being put forth throughout this country, to elevate our chosen profession, and establish an association of physicians whose united effort shall be to promote that *advancement* which is the spirit of the age."

The following are the officers of the Association:

*President.*—Dr. Samuel Denton, of Ann Arbor.

1*st Vice President.*—Dr. Geo. W. Gorham, of Jackson.

2*d Vice President.*—Dr. S. R. Arnold, of Monroe.

*Secretaries.*—Drs. DeLoskie and Miller, of Flint; and Geo. W. Fish, of Jackson.

*Treasurer.*—Dr. Abram Sager, of Ann Arbor.

Several Standing Committees were appointed to report at the next annual meeting, on practical medicine, surgery, obstetrics, and medical education. A special committee to report on the medical botany of the State was also appointed.

Appended to the proceedings are two addresses, one by the retiring President, Dr. Codman, of Lenawee, and the other by Dr. Field, of Adrian, formerly of N. Y., and now Demonstrator of Anatomy at the Geneva (New York) Medical College.

Both addresses are highly creditable productions. The work concludes with an extract of three pages from the sterling article on *Self-reformation of the Medical Profession*, by Prof. C. B. Coventry, in the March (1850) number of the Buffalo Medical Journal.

